# Inter-reader agreement and diagnostic accuracy of early postoperative magnetic resonance imaging for detecting macroscopic residual disease in neuroblastic tumors

**DOI:** 10.1007/s00247-026-06672-5

**Published:** 2026-05-28

**Authors:** Marianna Chaika, Cristian Urla, Steven W. Warmann, Michael Esser, Jakob Spogis, Patrick Krumm, Andreas Schmidt, Henrike Borkers, Thorsten Simon, Jörg Fuchs, Jürgen F. Schäfer

**Affiliations:** 1 Pediatric Radiology, Department of Radiology, University Hospital Tübingen, Tübingen, Germany; 2 Department of Pediatric Surgery and Pediatric Urology, University Children’s Hospital Tübingen, Tübingen, Germany; 3 Department of Pediatric Surgery, Charité - University Medicine Berlin, Berlin, Germany; 4 Department of Pediatric Hematology and Oncology, University Hospital Cologne, Cologne, Germany; 5 Diagnostic and Interventional Radiology, Universitätsklinikum Tübingen, Hoppe-Seyler-Str. 3, Tübingen, 72076 Germany

**Keywords:** Child, Magnetic resonance imaging, Neuroblastoma, Observer variation, Postoperative period, Residual neoplasm

## Abstract

**Background:**

Accurate detection of macroscopic residual disease after neuroblastoma resection is essential for postoperative treatment decisions, including radiotherapy. Standard imaging follow-up is often performed at a time point when postoperative and therapy-related changes may hamper reliable differentiation between residual disease and reactive findings.

**Objective:**

To assess inter-reader agreement and the diagnostic accuracy of early postoperative magnetic resonance imaging (MRI) for detecting residual disease after neuroblastoma surgery.

**Methods:**

This retrospective single-center study included patients with histologically confirmed neuroblastic tumors who underwent surgical resection at a reference center and received standardized early postoperative MRI with adequate preoperative imaging. Two independent pediatric radiologists, blinded to all clinical data, assessed all examinations. A hierarchical multimodal reference standard was established based on surgical reports including expert consensus between a senior pediatric radiologist and a pediatric surgeon, and follow-up imaging (median follow-up 33 months).

**Results:**

Thirty-nine patients (median age 46 months), predominantly with International Neuroblastoma Staging System (INSS) stage 4 neuroblastoma, were included; all patients had at least one image-defined risk factor. MRI was performed at a mean of 8±5 days after surgery. Residual disease was identified in 14 patients and confirmed by the reference standard. Five residual diseases were expected by the surgeons (median volume 8 ml), whereas nine were unexpected and small (median volume 1 ml). Diagnostic accuracy were 95% and 90% for the two readers, respectively, with substantial inter-reader agreement (Cohen’s* κ*=0.76). Early postoperative MRI findings led to clinically relevant management adaptations in selected cases.

**Conclusion:**

Early postoperative MRI demonstrates high diagnostic accuracy and substantial inter-reader agreement in detecting macroscopic residual disease, providing a robust baseline for further assessment.

**Graphical Abstract:**

**Figure:**
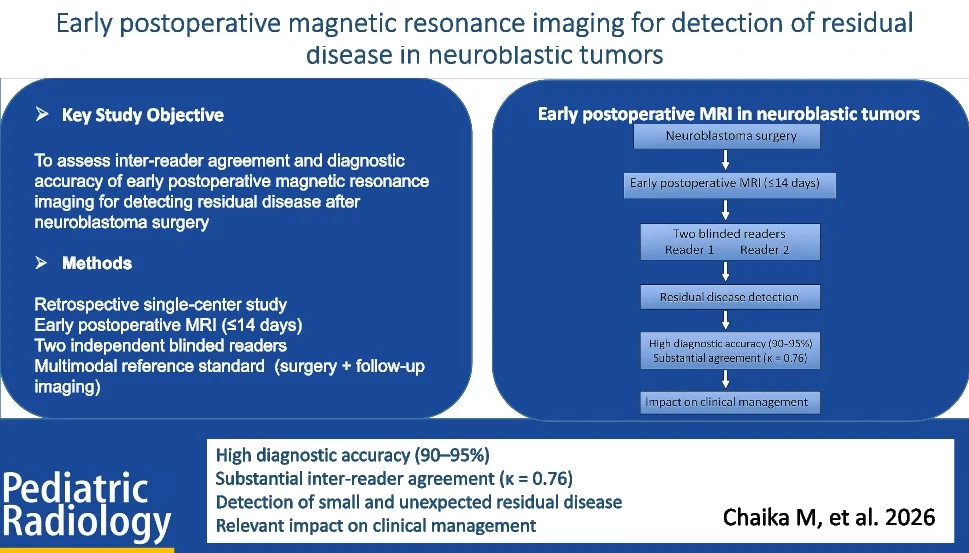

## Introduction

Neuroblastoma is the most common solid extracranial malignancy in children. Clinical, molecular-genetic, and image-defined risk factors determine the risk-adapted treatment, including chemotherapy, tumor resection, and radiotherapy [1]. Moreover, immunotherapy is an essential component of current treatment protocols [2]. Approximately 50% of patients are at high risk at the time of initial diagnosis [3]. Especially in abdominal midline tumors, incomplete macroscopic resection is not uncommon, as it aims to avoid postoperative complications or mutilations [3]. Microscopic residuals will be treated with tumor bed irradiation in high-risk patients [4, 5]. In cases of incomplete macroscopic resection, a boost therapy with 14.4 Gy is proposed [6, 7]. However, in former studies, this additional radiotherapy has not shown an overall benefit for event-free survival or overall survival (OS) [8]. In addition, especially in very young patients, therapy-associated toxicity is a limitation of this approach [9]. Some studies suggest that de-escalation of therapy may be possible in patients with tumor resection exceeding 90% [10]. Therefore, in the recent International Society of Paediatric Oncology Europe Neuroblastoma Group (SIOPEN) high-risk neuroblastoma 2 (HRNBL2) protocol, patients with macroscopic residual disease are randomized for additional boost. According to the protocol, residual disease is defined either by the surgeon’s report (macroscopic residual >5 ml) or by clear findings on postoperative imaging.

However, the first imaging time point is usually proposed to occur 3 months after resection. With this approach, it can be problematic to distinguish residual disease from scars, reactive changes, or early recurrence. Therefore, there is a discussion within oncology societies that are striving to introduce early postoperative MRI, similar to brain tumors [11]. In addition, differences between surgical and radiological reports regarding the presence of residual diseases may raise uncertainties. There is also an urgent need to understand the underlying causes of these discrepancies, which requires further research to develop more effective treatment approaches. Furthermore, a recently published study has shown high interobserver variability in determining the target volume contouring and dose planning of boost irradiation [12]. This result highlights the lack of precise diagnostic details for residual diseases on early MRI or computed tomography (CT) imaging.

Thus, this study aimed to analyze inter-reader agreement and diagnostic accuracy of an early MRI protocol after neuroblastoma resection to determine the extent of the remaining tumors. 

## Methods

### Patients and inclusion criteria

All pediatric patients assigned to our national Neuroblastoma reference center for surgical resection between June 2018 and October 2021 were reviewed. The inclusion criteria of this retrospective analysis were a histologically confirmed neuroblastic tumor, resection performed by the reference surgery, existence of preoperative MRI, and early postoperative MRI (maximum of 14 days after surgery) using the in-house standardized MRI protocol for neuroblastic tumors which is in concordance to the references [13–15].

All included cases were classified according to the INSS and also to the International Neuroblastoma Risk Group Staging System (INRGSS). The institutional ethical review board approved the retrospective study (No 814/2023BO2), and the requirement for informed consent was waived due to the retrospective study design.

### Magnetic resonance imaging protocol and residual disease detection

The MRI protocol for staging and restaging neuroblastoma included the following sequences: coronal T2w, transverse T1w vibe Dixon before and after contrast, transversal T2-weighted sequences with fat saturation with navigator technique and radial k-space sampling if available, and transversal diffusion-weighted imaging (DWI) with the calculation of the apparent diffusion coefficient (ADC) map (Table 1). In addition, an extension of the protocol was adapted to the clinical needs and questions (e.g., MR-angiography, additional planes, or whole-body sequences). Patients under the age of 5 years received general anesthesia for the examination.

**Table 1:** Basic magnetic resonance imaging sequences for assessment of pre- and postoperative tumor load

Basic sequence type	Plane	Pixel spacing [mm2]	Slice thickness [mm]	Comments
T2w TSE	Coronal	1.25	3	Option HASTE, respiratory triggering
T2w TSE, fat sat	Transversal	0.625–0.8	4	Radial sampling and respiratory triggering
T1w Vibe Dixon	Transversal	1.0	2–3	If Dixon not available T1 with and without fs. Option radial sampling
T1w Vibe Dixon pCM	Transversal	1.0	2–3	If Dixon not available T1 with fs. Option radial sampling
DWI	Transversal	1.0	3	Minimal two *b*-values, ADC calc

*TSE* turbo spin echo, *Vibe* volume interpolated breath-hold imaging, *DWI* diffusion-weighted imaging, *HASTE* half-Fourier acquisition single-shot turbo spin echo

Image analysis was performed by two readers: a board-certified pediatric radiologist M.E. with 7 years of experience in pediatric imaging (R1), and a radiologist J.S. in advanced subspecialty training in pediatric radiology with 2 years of dedicated experience (R2). Both readers were wholly independent and blinded to patients’ characteristics, surgical reports, follow-up examinations, and clinical outcomes. Only the two MRI examinations (before and after surgery) were available. The presence and localization of the residual disease were recorded. Residual disease was defined as noticeable tissue at the exact location, with comparable T1 and T2 signal intensities, and ADC values similar to those of the primary tumor, as observed in the preoperative imaging. ADC values were assessed using reader-defined regions of interest (ROIs) placed within the suspected residual disease to sample representative areas. Lesion detection and characterization were both part of the blinded reader assessment and included in the calculation of diagnostic accuracy.

### Comprehensive analysis

A radiology resident first delineated all areas potentially indicative of residual disease on the postoperative MRI using a volume of interest (VOI)-based approach on a dedicated post-processing workstation (syngo.via, Siemens Healthineers, Erlangen, Germany). Next, these segmentations were verified by an independent senior pediatric radiologist. This verification step ensured technical consistency of the segmentation and volumetric assessment, but did not involve diagnostic interpretation. Notably, this senior radiologist did not serve as one of the two blinded study readers.

For clinical determination of macroscopic residual disease, a hierarchical multimodal reference standard was defined. An independent senior pediatric radiologist (the same individual who had approved the segmentation, but not a reader) and an independent senior pediatric surgeon jointly re-evaluated all available operative information, intraoperative photographs, and the pre- and a minimum of two postoperative MRI examinations. Longitudinal follow-up imaging and clinical data were incorporated. Follow-up information was traceable for all with a median observation time of 33 months (95% CI 22 months to 45 months). Operative reports, which varied in descriptive precision, were categorized as complete macroscopic excision or incomplete macroscopic excision according to established criteria [16, 17]. Histological confirmation of residual disease was not pursued, as re-resection or biopsy would not be ethically justified and is not recommended in international neuroblastoma guidelines. Final classification as residual disease or no residual disease was determined by consensus between the senior pediatric radiologist and senior pediatric surgeon, integrating all available surgical, imaging, and follow-up data. False-negative findings were defined as residual disease present according to the reference standard but not detected by the reader, whereas false-positive findings were defined as suspected residual disease on MRI not confirmed by the reference standard.

### Statistical analysis

Data analysis was performed using JMP 16 (SAS Institute, Cary, NC, USA) and MedCalc version 18.1 (MedCalc Software, Ostend, Belgium). Normality of data distribution was assessed using the Shapiro-Wilk test, and mean or median values were reported accordingly. Sensitivity, specificity, and accuracy were calculated, with corresponding 95% confidence intervals. Cohen’s kappa was used to evaluate inter-rater reliability. Statistical significance was determined at a threshold of *P* < 0.05.

## Results

### Patients’ characteristics

Of 109 patients with neuroblastic tumors who underwent surgery between 2018 and 2021, a total of 39 patients (21 female) with a median age of 46 months at the time of surgery (IQR 54, range 5–177) met all inclusion criteria. The main reasons for exclusion were the absence of early postoperative MRI, particularly in less complex cases (e.g., stage L1), and insufficient quality of externally acquired preoperative MRI, which in some cases led to the use of CT imaging instead. Eleven patients had MYCN-amplified tumors, four were 1p36 positive, and two were ALK-amplification positive. The mIBG status was unavailable in some cases.

According to the INSS, 34 cases had stage 4, and five cases had stage 3. All included patients had image-defined risk factors according to the INRGSS, indicating intermediate- or high-risk disease; low-risk cases were not part of the study cohort. The final histopathological diagnosis of the specimen (not of the biopsy before treatment) was neuroblastoma in 23 cases, ganglioneuroblastoma in 13 cases, and ganglioneuroma in three cases, reflecting post-treatment tumor maturation rather than the initial risk classification. This finding is consistent with therapy-induced maturation from neuroblastoma to ganglioneuroblastoma, reflecting the known histopathological heterogeneity and mixed composition of neuroblastic tumors. Preoperative MRI examinations were performed at a median of 14 days (1–90 days) before surgery, and postoperative MRI was performed at a median of 8 (+/—5) days after surgery. The patients’ characteristics are shown in Table 2.

**Table 2:** Patients’ characteristics

Patients	39
Sex	18 male, 21 female
Mean age ± SD	52±38 months (range 5–177 months)
INSS	34 cases with stage 4, and 5 cases with stage 3
Neuroblastoma	*n*=23
Ganglioneuroblastoma	*n*=13
Ganglioneuroma	*n*=3
MYCN-positive/negative/unavailable	*n*=11/26/2
ALK-mutation-positive	*n*=2
1p36 aberration positive	*n*=4

**Fig. 1.**
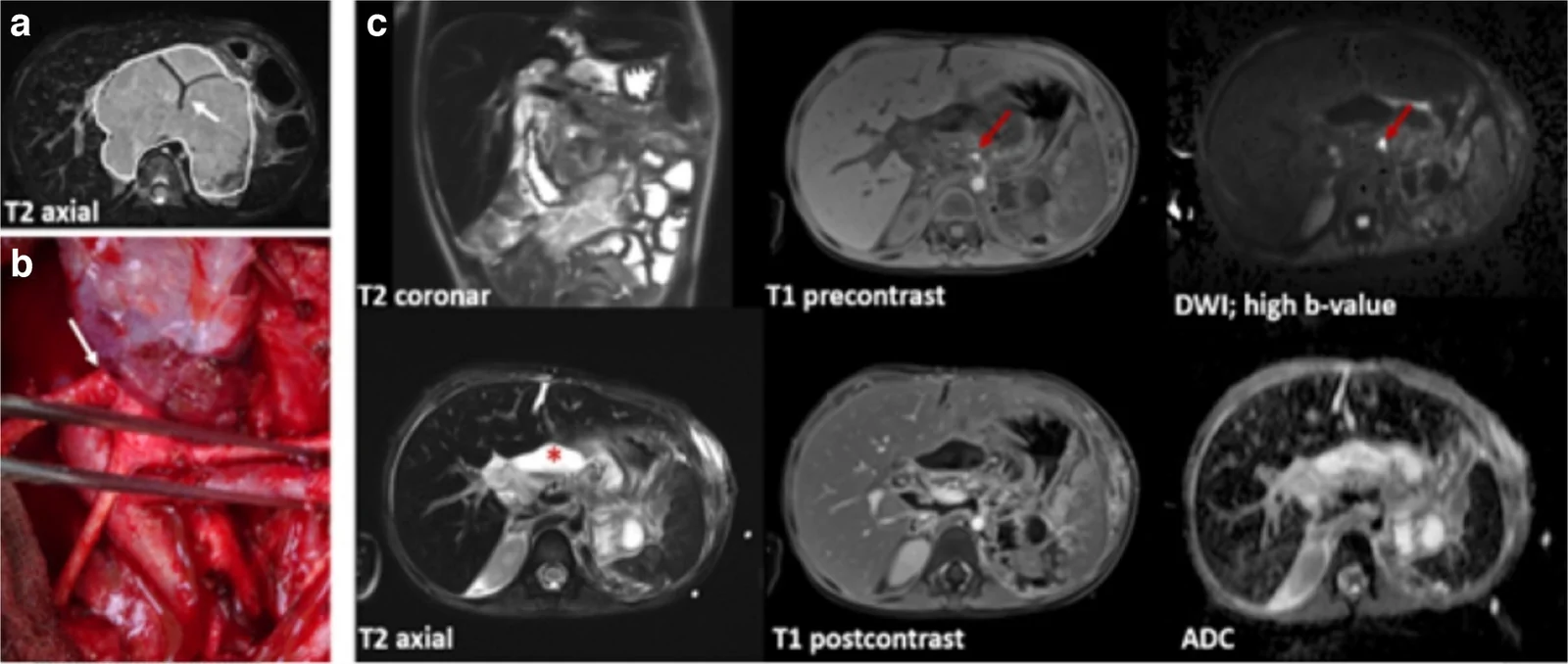
A 2-year-old female patient. **a** Preoperative magnetic resonance imaging shows a large midline neuroblastoma at the level of the celiac trunk (*white arrow*). **b** Intraoperative photograph demonstrates macroscopic complete excision (*white arrow*). **c** Early postoperative magnetic resonance imaging (day 10) shows fluid in the resection area (*red star*) and postoperative hemorrhage without evidence of residual disease (*red arrows*)

**Fig. 2.**
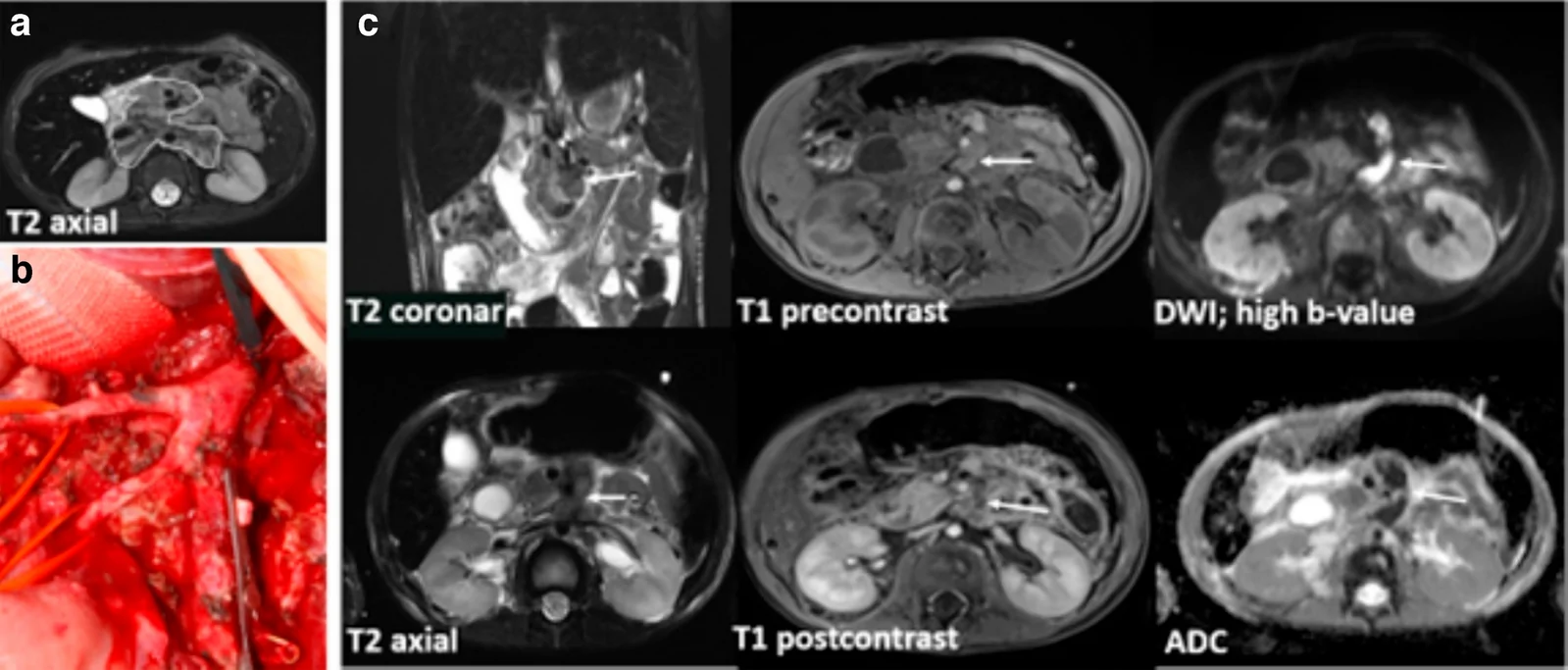
A 5-year-old female patient. **a** Preoperative magnetic resonance imaging performed 6 days after surgery shows a retroperitoneal neuroblastoma (*cursive*). **b** Intraoperative photograph demonstrates no visible residual disease in the resection area. **c** Early postoperative magnetic resonance imaging shows residual tumor adjacent to the duodenum (*arrows*). This discrepancy can be explained by the deep anatomical location of the tumor in the mesenteric root close to major vascular structures, limiting intraoperative visualization

**Fig. 3.**
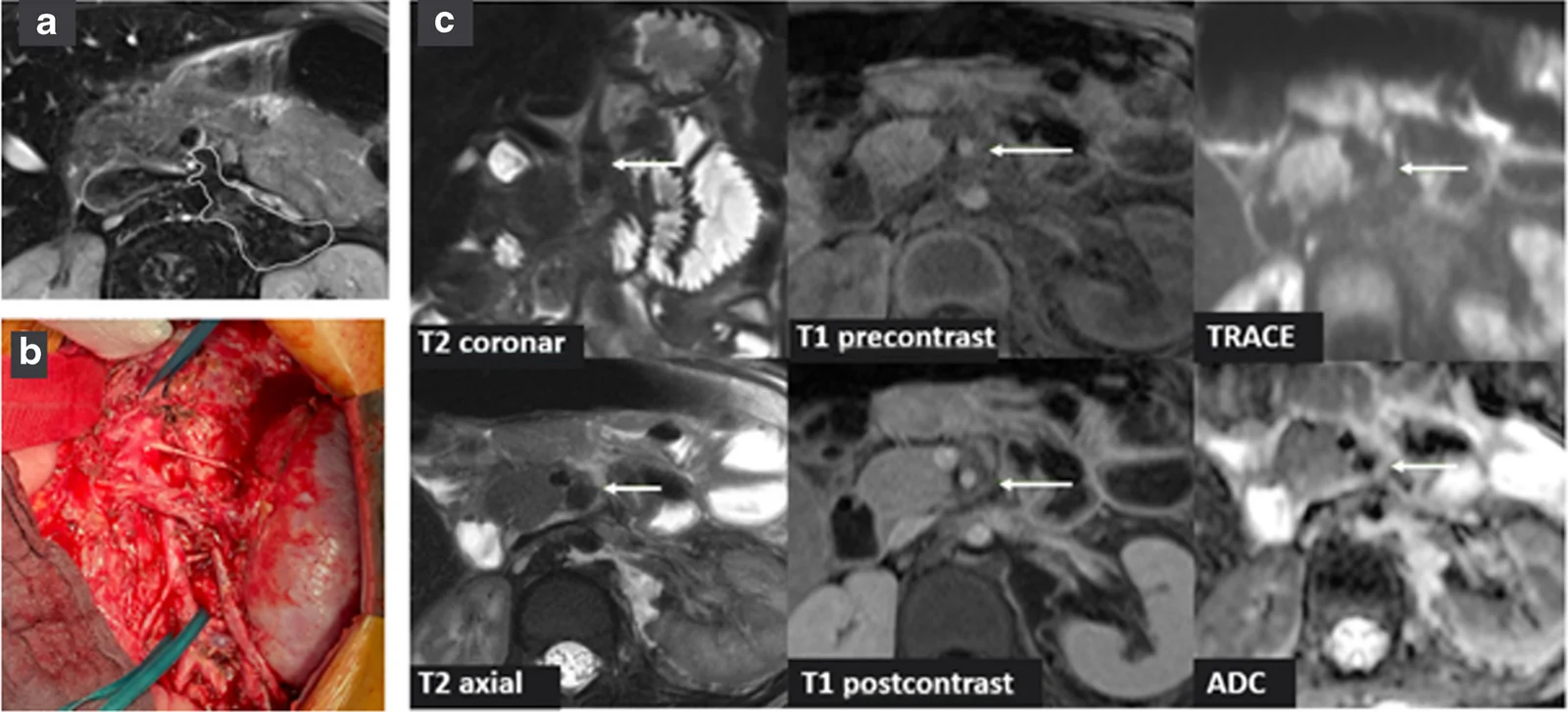
A 5-year-old female patient. **a** Preoperative T2-weighted magnetic resonance imaging shows neuroblastoma (*cursive*) encasing the superior mesenteric artery (*asterisk*). **b** Intraoperative image demonstrates no visible residual disease. **c** Early postoperative magnetic resonance imaging shows subtle residual tumor as eccentric tissue thickening around the superior mesenteric artery (*arrows*). This lesion was not identified by either reader and was classified as a false-negative finding

According to the surgical reports, complete macroscopic excision was achieved in 34 of 39 patients. All tumors were resected using an open surgical approach, consistent with the presence of multiple image-defined risk factors in this cohort. No relevant differences in patient or tumor characteristics were observed between cases with complete and incomplete macroscopic resection. The comprehensive analysis finally confirmed residual diseases in 14 patients, most often with a location at the mesenteric root and retro-crural. Five residual diseases were documented by the surgeons with a median volume in MRI of 8 ml (IQR 1–30). Nine residual diseases were not documented, with a median MRI volume of 1 ml (IQR 0.5–1.75, *P*=0.14). The readers had 37 (R1) and 35 (R2) of 39 cases, which were true positives or negatives. Thus, the accuracies were 95% (R1) and 90% (R2). Reader 1 rated one case as a false negative and one case as a false positive, and reader 2 rated three cases as false negatives and one case as a false positive (Table 3). The volume of the false-negatively rated cases (*n*=4) ranged from 0.2 ml to 0.5 ml. The inter-rater agreement was substantial (Cohen’s kappa 0.76). Table 4 shows the cases for both readers that were discrepant with the reference standard. Figures 1, 2, and 3 show examples of discrepancies between the readers and the reference standard.

**Table 3:** Residual disease detectability by two readers

Detectability	Reader 1	Reader 2
True positive	13	11
True negative	24	24
False positive	1	1
False negative	1	3
Sensitivity	0.93 (CI 95%, 0.69 to 0.99)	0.79 (CI 95%, 0.52 to 0.92)
Specificity	0.96 (CI 95%, 0.80 to 0.99)	0.96 (CI 95%, 0.80 to 0.99)
Accuracy	0.95 (CI 95%, 0.83 to 0.99)	0.90 (CI 95%, 0.76 to 0.96)
Cohen’s kappa	0.77 (CI 95%, 0.56 to 0.98)

**Table 4:** Discrepancy regarding residual disease identification and localization between the reference and the readers

Post-OP MRI, day	Reference	Reader 1	Reader 2
	Location and volume	Location	
9	Residual disease at the mesenteric root, 0.5 ml	Residual disease at the mesenteric root	No residual disease
3	Right retro-crural, 0.2 ml	Right paravertebral	No residual disease
0	No residual disease	No residual disease	Residual disease retroperitoneal
8	Right adrenal 0.5 ml	No residual disease	No residual disease
7	No residual disease	Left adrenal	No residual disease

### Postoperative complications and follow-up findings

After resection of neuroblastic tumors, the following complications were observed by early postoperative MRI: hematomas in eight patients, arterial stenosis in five patients, intravenous thrombi in two, abscesses in two, ileus in one patient, inflammation of the renal pelvis, and a large amount of ascites in three patients. According to the Dindo-Clavien grading, four complications were classified as grade IIIb, one as grade II, and 12 as grade I [17]. Prompt re-laparotomy was performed for four complications: acute bleeding, thrombosis of the renal vein, stenosis of the celiac trunk, and ileus. In two patients with pronounced ascites, parenteral nutrition was indicated, and antibiotic treatment was given for inflammation of the renal pelvis. Furthermore, re-operation after early postoperative MRI was indicated due to an overseen adrenal tumor following laparoscopic resection. In two cases with negative results in the early MRI, the second MRI after surgery demonstrated reactive lymph node swelling again confirmed by positron emission tomography (PET) and follow-up. In one case, a new lesion was seen in the resection area after 3 months; thus, an early recurrence was diagnosed.

## Discussion

Early postoperative MRI proved highly valuable for the precise identification and quantification of macroscopic residual disease, demonstrating high diagnostic accuracy and consistent agreement between experienced readers, as well as for the detection of perioperative complications. Determining residual disease shortly after surgery increases diagnostic confidence and directly informs subsequent imaging strategies, radiotherapy planning, and systemic treatment decisions. Early MRI is particularly informative in unexpectedly small residual lesions that are not visible intraoperatively, highlighting the added value of imaging beyond surgical inspection.

Distinguishing residual disease from postoperative changes remains challenging, especially because residual disease frequently presents with heterogeneous signal characteristics [18]. Accordingly, our protocol-aligned with the forthcoming SIOPEN imaging recommendations-combines T2-weighted sequences with and without fat saturation, Dixon-based pre- and post-contrast T1-weighted imaging, and diffusion-weighted imaging with ADC mapping. The Dixon sequence aids differentiation between residual disease, fluid, and hemorrhage, whereas non–fat-suppressed sequences facilitate the detection of low-signal regressive components. DWI was particularly helpful in detecting small-volume residuals and distinguishing maturation phenomena of neuroblastic tumors [19–21]. The relatively short acquisition time of the protocol is crucial for postoperative pediatric patients, who are usually referred from the intensive care unit prior to transfer to the regular ward. In addition, this protocol is expected to be useful for intraoperative MRI, which represents a potential future application.

The diagnostic robustness of early postoperative MRI in identifying macroscopic residual disease is fundamentally based on the excellent comparability of neuroblastic tumor characteristics before and after surgery. As demonstrated in earlier studies, MRI provides highly reliable assessment of preoperative tumor extent and tissue properties, including T1- and T2-based morphology, contrast enhancement patterns, and quantitative ADC behavior [22–24] In accordance with these principles, residual disease in our study was defined as tissue displaying the same qualitative signal profile and diffusion characteristics as the preoperative mass at the identical anatomical location.

The diagnostic accuracy of both pediatric radiologists was excellent (95% and 90%), despite the restriction to only two imaging time points. False-negative cases were extremely small (0.2–0.5 ml), underscoring the high sensitivity of the protocol even for sub-centimeter lesions. Blinding the readers to operative reports—contrary to real-world clinical practice—was methodologically essential to avoid incorporation bias. Inter-reader agreement was substantial, indicating robust detectability of residual disease within a pediatric radiology expertise framework. Overall, the findings underscore the importance of radiological expertise in neuroblastoma imaging, particularly for subtle tissue differences [25]. However, the influence of reader experience and image quality was not specifically analyzed in this study. This specialized experience is key for reliably identifying residual disease in children and guiding appropriate follow-up therapy.

Importantly, early postoperative MRI provides an essential baseline that supports the differentiation between residual tumor, postoperative reactive changes, and true recurrence during follow-up. In two patients, reactive lymph node enlargement detected on later follow-up imaging mimicked recurrence; however, early postoperative MRI had documented the absence of residual tumor, thereby facilitating more accurate interpretation of subsequent findings. Conversely, in one case, new tissue identified in the resection bed 3 months after surgery represented early recurrence rather than postoperative changes, a distinction made possible by comparison with the early postoperative MRI as baseline. In addition, early postoperative MRI directly influenced clinical management: an overlooked adrenal tumor following laparoscopic resection was detected, prompting immediate re-operation. These cases illustrate that early postoperative MRI does not replace follow-up imaging but rather provides the critical reference framework that prevents both under- and overtreatment. Furthermore, it enables a more reliable distinction between residual disease, postoperative changes, and early recurrence in longitudinal assessment.

The hierarchical comprehensive analysis-combining an independent expert review with long-term follow-up (median 33 months)-enhanced the validity of the ground truth and minimized verification bias. This mirrors established clinical practice in neuroblastoma reference centers, where postoperative MRI is interpreted jointly by experienced pediatric radiologists and surgeons and subsequently contextualized with longitudinal disease evolution. Such an approach compensates for the limitations of operative inspection alone, particularly in anatomically concealed regions such as the retro-crural space or mesenteric branches. In this context, residual diseases not documented by the surgeons were observed relatively often (9 of 13), typically with small volumes and in locations known to be challenging or impossible to resect. All patients had at least one image-defined risk factor, reflecting the complexity of their surgical anatomy. Surgical inexperience as a confounding factor can be excluded, as all procedures were performed by two senior pediatric surgeons in a national reference center. 

The role of surgery and the effects of complete macroscopic excision vs. incomplete macroscopic excision are still under discussion [26]. Notably, Holmes et al., who relied exclusively on surgeons’ documentation, demonstrated in their study that incomplete removal of tumors is associated with a higher risk of disease progression and recurrence [16]. This finding was not confirmed, for example, by the analysis of the GPOH NB97 study, which was based on a comprehensive evaluation of surgeons’ reports and postoperative imaging [27]. Another recent study has demonstrated that residual disease detected by MRI was associated with relapses or local progression in 50% of patients [15]. Current Gesellschaft für Pädiatrische Onkologie und Hämatologie (GPOH) practice, supported by inclusion in the guidelines of the recently completed Children’s Oncology Group (COG) phase 3 high-risk neuroblastoma trial ANBL0532, shifted to treating unresected gross residual disease with a 14.4-Gy boost [10, 28]. In the Chicago Pilot II protocol as well as in the SIOPEN HR NBL1 (ClinicalTrials.gov ID NCT01704716 and ClinicalTrials.gov ID NCT04221035) trials, patients with high-risk neuroblastoma are subject to receive radiation to the primary tumor site regardless of the extent of resection [16, 29]. Ferris et al. have demonstrated no disadvantage of radiation for gross residual disease [30]. Nevertheless, numerous studies have shown conflicting results, highlighting both the potential disadvantages and benefits of postoperative radiotherapy. For example, Lucas et al. suggested that de-escalation of radiotherapy is possible in patients with more than 90% tumor resection and without primary image-defined risk factor [31]. Liu et al. have also shown that boost radiotherapy did not lessen local progression in the presence of marked residual disease [8]. Therefore, improving the diagnostic accuracy of early MRI scans can potentially reduce unnecessary radiation exposure in pediatric patients without residual disease.

The early-postoperative MRI protocol is important not only for the question of the residual disease but also for the entire diagnosis in the postoperative complications, correlation with the surgical report, e.g., image-defined risk factor, follow-up, and assessment of remission status [2]. Although many postoperative complications can also be detected by CT, MRI was primarily used in this study for residual tumor assessment and baseline definition, while avoiding ionizing radiation. It has been shown that postoperative complications were detected in the early-postoperative MRI, where in four cases, a prompt re-laparotomy was needed. This fact suggests that an early-postoperative MRI should be performed early after surgery.

The implementation of early postoperative MRI in routine clinical practice entails relevant economic, logistical, and anesthesiological considerations, particularly in younger children who may require sedation or general anesthesia. This approach requires additional resources and infrastructure that may not be readily available at all centers. However, a comprehensive assessment should also consider the potential benefits, including avoiding unnecessary treatments such as radiation therapy and its associated long-term sequelae.

Our study has several limitations: First, the sample size is relatively small, as only approximately 35% of the patients who underwent resection were included. The main reasons for exclusion were the lack of early postoperative MRI, particularly in less complex cases (e.g., stage L1), and insufficient quality of externally acquired preoperative MRI. In such cases, CT imaging was often performed instead due to better availability. Therefore, a selection bias cannot be completely ruled out. Second, the study is a single-center experience with a retrospective design. inter-reader agreement was assessed between two blinded readers using Cohen’s kappa, the standard and widely accepted measure of inter-reader reliability. Additional readers may improve generalizability, but are not required for a valid assessment of agreement. Thus, the number of readers mirrors clinical practice and does not indicate a methodological limitation of the imaging approach. A potential recall bias cannot be fully excluded, but is considered unlikely due to the retrospective design and independent second reader.

In conclusion, accurate assessment of macroscopic residual disease is essential for prognostic stratification and postoperative treatment planning in neuroblastic tumors. Using an adapted early-postoperative MRI protocol, image interpretation by experienced pediatric radiologists yielded reliable results with substantial inter-reader agreement, even in a limited patient cohort. Importantly, early-postoperative MRI provided a robust baseline for follow-up assessment and led to clinically relevant management changes in selected cases, including re-operation and refinement of further treatment strategies. These findings support the value of early-postoperative MRI as a complementary component of postoperative evaluation in specialized reference-center settings. Furthermore, early-postoperative MRI can provide a reliable baseline for follow-up imaging and inform clinical decisions; however, its effect on patient outcomes warrants further study.

## Data Availability

Not applicable.

## References

[1] Avanzini S, Pio L, Erminio G et al (2017) Image-defined risk factors in unresectable neuroblastoma: SIOPEN study on incidence, chemotherapy-induced variation, and impact on surgical outcomes. Pediatr Blood Cancer 64 10.1002/pbc.26605 28440012

[2] Yu AL, Gilman AL, Ozkaynak MF et al (2010) Anti-GD2 antibody with GM-CSF, interleukin-2, and isotretinoin for neuroblastoma. N Engl J Med 363:1324–1334 10.1056/NEJMoa0911123 PMC3086629 20879881

[3] Qi Y, Zhan J (2021) Roles of surgery in the treatment of patients with high-risk neuroblastoma in the Children Oncology Group study: a systematic review and meta-analysis. Front Pediatr 9:706800 34722415 10.3389/fped.2021.706800 PMC8548868

[4] Fischer J, Pohl A, Volland R et al (2017) Complete surgical resection improves outcome in INRG high-risk patients with localized neuroblastoma older than 18 months. BMC Cancer 17:520 10.1186/s12885-017-3493-0 PMC5543757 28778185

[5] Seemann NM, Erker C, Irwin MS et al (2023) Survival effect of complete surgical resection of the primary tumor inpatients with metastatic, high-risk neuroblastoma in a large Canadian cohort. Pediatr Blood Cancer 70 10.1002/pbc.30286 36975166

[6] Bradfield SM, Douglas JG, Hawkins DS et al (2004) Fractionated low-dose radiotherapy after myeloablative stem cell transplantation for local control in patients with high-risk neuroblastoma. Cancer 100:1268–1275 10.1002/cncr.20091 15022296

[7] Gatcombe HG, Marcus RB Jr., Katzenstein HM et al (2009) Excellent local control from radiation therapy for high-risk neuroblastoma. Int J Radiat Oncol Biol Phys 74:1549–1554 10.1016/j.ijrobp.2008.10.069 19211198

[8] Liu KX, Naranjo A, Zhang FF et al (2020) Prospective evaluation of radiation dose escalation in patients with high-risk neuroblastoma and gross residual disease after surgery: a report from the Children’s Oncology Group ANBL0532 Study. J Clin Oncol 38:2741–2752 10.1200/JCO.19.03316 PMC7430214 32530765

[9] Zhao Q, Liu Y, Zhang Y et al (2020) Role and toxicity of radiation therapy in neuroblastoma patients: a literature review. Crit Rev Oncol Hematol 149:102924 10.1016/j.critrevonc.2020.102924 32172225

[10] von Allmen D, Davidoff AM, London WB et al (2017) Impact of extent of resection on local control and survival in patients from the COG A3973 study with high-risk neuroblastoma. J Clin Oncol 35:208–216 10.1200/JCO.2016.67.2642 PMC5455676 27870572

[11] Kamp MA, Rapp M, Buhner J et al (2015) Early postoperative magnetic resonance tomography after resection of cerebral metastases. Acta Neurochir (Wien) 157:1573–1580 10.1007/s00701-015-2479-4 26156037

[12] Jazmati D, Brualla L, Littooij AS et al (2023) Overcoming inter-observer planning variability in target volume contouring and dose planning for high-risk neuroblastoma - a European multicenter effort of the SIOPEN radiotherapy committee. Radiother Oncol 181:109464 10.1016/j.radonc.2023.109464 36640946

[13] Sarioglu FC, Salman M, Guleryuz H et al (2019) Radiological staging in neuroblastoma: computed tomography or magnetic resonance imaging? Pol J Radiol 84:e46–e53 10.5114/pjr.2019.82736 PMC6479053 31019594

[14] Chen AM, Trout AT, Towbin AJ (2018) A review of neuroblastoma image-defined risk factors on magnetic resonance imaging. Pediatr Radiol 48:1337–1347 30078048 10.1007/s00247-018-4117-9

[15] Schafer JF, Gassenmaier S, Warmann S et al (2023) Local MRI before and after tumor resection in neuroblastoma: impact of residual disease on event free survival. J Clin Med 12:2338 10.3390/jcm12237297 PMC10707530 38068349

[16] Holmes K, Potschger U, Pearson ADJ et al (2020) Influence of surgical excision on the survival of patients with stage 4 high-risk neuroblastoma: a report from the HR-NBL1/SIOPEN Study. J Clin Oncol 38:2902–2915 10.1200/JCO.19.03117 32639845

[17] La Quaglia MP, Kushner BH, Su W et al (2004) The impact of gross total resection on local control and survival in high-risk neuroblastoma. J Pediatr Surg 39:412–417 discussion 412–417 10.1016/j.jpedsurg.2003.11.028 15017562

[18] Park JR, Bagatell R, Cohn SL et al (2017) Revisions to the international neuroblastoma response criteria: a consensus statement from the National Cancer Institute Clinical Trials Planning Meeting. J Clin Oncol 35:2580–2587 10.1200/JCO.2016.72.0177 PMC5676955 28471719

[19] Gahr N, Darge K, Hahn G et al (2011) Diffusion-weighted MRI for differentiation of neuroblastoma and ganglioneuroblastoma/ganglioneuroma. Eur J Radiol 79:443–446 10.1016/j.ejrad.2010.04.005 20462716

[20] Gassenmaier S, Tsiflikas I, Fuchs J et al (2020) Feasibility and possible value of quantitative semi-automated diffusion weighted imaging volumetry of neuroblastic tumors. Cancer Imaging 20:89 10.1186/s40644-020-00366-3 PMC7745476 33334369

[21] Neubauer H, Li M, Muller VR et al (2017) Diagnostic value of diffusion-weighted MRI for tumor characterization, differentiation and monitoring in pediatric patients with neuroblastic tumors. Rofo 189:640–650 10.1055/s-0043-108993 28511265

[22] Littooij AS, de Keizer B (2023) Imaging in neuroblastoma. Pediatr Radiol 53:783–787 36063183 10.1007/s00247-022-05489-2 PMC10027638

[23] Voss SD (2018) Staging and following common pediatric malignancies: MRI versus CT versus functional imaging. Pediatr Radiol 48:1324–1336 30078040 10.1007/s00247-018-4162-4

[24] Gassenmaier S, Tsiflikas I, Maennlin S et al (2020) Retrospective accuracy analysis of MRI based lesion size measurement in neuroblastic tumors: which sequence should we choose? BMC Med Imaging 20:105 10.1186/s12880-020-00503-1 PMC7487996 32912148

[25] Thukral BB (2015) Problems and preferences in pediatric imaging. Indian J Radiol Imaging 25:359–364 26752721 10.4103/0971-3026.169466 PMC4693383

[26] Zwaveling S, Tytgat GA, van der Zee DC et al (2012) Is complete surgical resection of stage 4 neuroblastoma a prerequisite for optimal survival or may >95 % tumour resection suffice? Pediatr Surg Int 28:953–959 10.1007/s00383-012-3109-3 PMC3445800 22722825

[27] Simon T, Häberle B, Hero B et al (2013) Role of surgery in the treatment of patients with stage 4 neuroblastoma age 18 months or older at diagnosis. J Clin Oncol 31:752–758 10.1200/JCO.2012.45.9339 23284039

[28] Simon T, Thole T, Castelli S et al (2025) GPOH guidelines for diagnosis and first-line treatment of patients with neuroblastic tumors, update 2025. Klin Padiatr 237:117–140 10.1055/a-2556-4302 40345224

[29] Wolden SL, Gollamudi SV, Kushner BH et al (2000) Local control with multimodality therapy for stage 4 neuroblastoma. Int J Radiat Oncol Biol Phys 46:969–974 10.1016/s0360-3016(99)00399-5 10705019

[30] Ferris MJ, Danish H, Switchenko JM et al (2017) Favorable local control from consolidative radiation therapy in high-risk neuroblastoma despite gross residual disease, positive margins, or nodal involvement. Int J Radiat Oncol Biol Phys 97:806–812 10.1016/j.ijrobp.2016.11.043 PMC5502807 28244417

[31] Lucas JT Jr., McCarville MB, Cooper DA et al (2019) Implications of image-defined risk factors and primary-site response on local control and radiation treatment delivery in the management of high-risk neuroblastoma: is there a role for de-escalation of adjuvant primary-site radiation therapy? Int J Radiat Oncol Biol Phys 103:869–877 10.1016/j.ijrobp.2018.11.041 PMC8810202 30496881

